# Geometrically Controlled Microscale Patterning and Epitaxial Lateral Overgrowth of Nitrogen-Polar GaN

**DOI:** 10.1021/acs.cgd.4c00235

**Published:** 2024-07-30

**Authors:** Pietro Pampili, Vitaly Z. Zubialevich, Peter J. Parbrook

**Affiliations:** † Tyndall National Institute, University College Cork, Lee Maltings Dyke Parade, Cork T12 R5CP, Ireland; ‡ School of Engineering, University College Cork, Western Road, Cork, Ireland

## Abstract

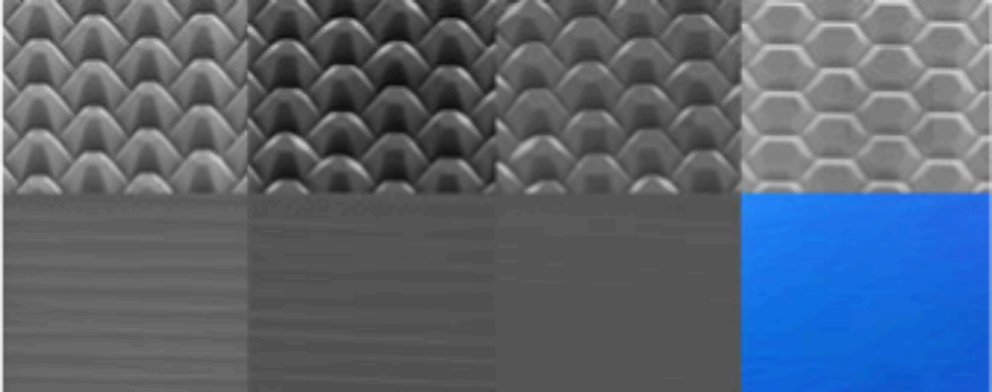

In this Letter, we report on a novel two-step epitaxial growth technique that enables a significant improvement of the crystal quality of nitrogen-polar GaN. The starting material is grown on 4° vicinal sapphire substrates by metal-organic vapor-phase epitaxy, with an initial high-temperature sapphire nitridation to control polarity. The material is then converted to a regular array of hexagonal pyramids by wet-etching in a KOH solution and subsequently regrown to coalesce the pyramids back into a smooth layer of improved crystal quality. The key points that enable this technique are the control of the array geometry, obtained by exploiting the anisotropic behavior of the wet-etch step, and the use of regrowth conditions that preserve the orientation of the pyramids’ sidewalls. In contrast, growth conditions that cause an excessive expansion of the residual (0001̅) facets on the pyramids’ tops cause the onset of a very rough surface morphology upon full coalescence. An X-ray diffraction study confirms the reduction of the threading dislocation density as the regrowth step develops. The analysis of the relative position of the 0002̅ GaN peak, with respect to the 0006 sapphire peak, reveals a macroscopic tilt of the pyramids, probably induced by the large off-axis substrate orientation. This tilt correlates very well with an anomalous broadening of the 0002̅ diffraction peaks at the beginning of the regrowth step.

## Abstract 2

N-polar GaN is a material of high technological relevance, especially for applications in the domain of high-frequency transistors. Unfortunately, compared to standard Ga-polar GaN, the growth of high-quality epilayers is much more difficult. In this study, we report about a novel procedure based on epitaxial overgrowth of patterned N-polar GaN templates that allows a large improvement in crystal quality.

The growth of N-polar GaN has been intensively studied over the last two decades, especially for application in the field of high-frequency electronics, due to the disruptive approach in transistor scaling that N-polar III nitrides enable.^1^ It is now well understood that N-polarity can be controlled either by using the appropriate face of a polar substrate, such as the carbon face of SiC, or by applying a pregrowth high-temperature nitridation step to a sapphire substrate.^2^ Early N-polar samples showed very rough surfaces, which was understood as due to reduced adatom mobility on N-polar surfaces and consequent random nucleation of hexagonal islands. An important breakthrough was the demonstration of smooth surface morphologies, which was made possible by the use of vicinal substrates with offcut angles as large as 4°.^3,4^

However, despite all these achievements, the crystal quality of N-polar GaN is still somewhat poorer than that of standard Ga-polar epilayers, with X-ray rocking curve full width at half maxima (fwhm) values that are ∼400 and ∼600 arcsec for symmetric (e.g., 0002̅) and skew-symmetric (e.g., 101̅2̅ and 101̅1̅) reflections, respectively, for most of the best samples reported in the literature (see, for example, ref (4) for sapphire and ref (5) for SiC substrates; more references can be found also in ref (1)).

In the past, several growth techniques have been developed to reduce the threading dislocation density of heteroepitaxial films. An effective approach is based on the initial formation of microscale structures having slanted sidewalls, followed by the subsequent coalescence of these structures into a smooth, higher-quality epilayer. During the coalescence phase, the dislocations that cross any of the slated facets are forced to bend outward, which increases the likelihood of meeting and annihilating with other nearby dislocations. Examples of this approach in III nitrides are the Facet Initiated Epitaxial Lateral Overgrowth (FIELO) technique, whereby slanted sidewalls occur spontaneously with hydride vapor phase epitaxy (HVPE),^6^ and the Facet Controlled Epitaxial Lateral Overgrowth (FACELO) technique, whereby specific growth conditions are needed to induce slanted sidewalls in metal-organic vapor-phase epitaxy (MOVPE).^7^ We also developed a similar approach with lateral expansion of nanocolumns.^8^ Despite the evident success of this approach for Ga-polar materials, there is currently no report of similar techniques for N-polar materials, which is the topic of this Letter.

A striking difference between Ga- and N-polar GaN is that, while the former is chemically very stable, the latter can be easily wet-etched in KOH or other hydroxide-containing solutions, which makes this the simplest and most widely used way to discriminate between the two polarities.^9^ It is also well-known that, during etching, the (0001̅) face quickly converts to a random distribution of hexagonal pyramids with slanted facets oriented along the {11̅01̅} planes, a fact that has been used, for example, to roughen the bottom N-polar face of laser-lifted-off LEDs to increase light-extraction efficiency.^10^ These naturally occurring structures would be perfect candidates for a FACELO-like technique for N-polar materials, with the additional advantage of not requiring any particular optimization of the growth conditions to force their appearance. Wet etching is also a very simple and low-cost procedure that does not induce crystal damage, as opposed to plasma-based dry etching.^11^ Unfortunately, the fact that the pyramids form with a large distribution of heights^10,12^ makes it virtually impossible to convert them back to a smooth epilayer during the coalescence phase. Hence, the development of a new strategy for controlling the pyramid geometry during etching is crucial.

From the observation that the wet-etch of N-polar materials continues until the epilayer is completely dissolved and does not stop even after there are no more facets left and the surface is fully covered in pyramids, it is often concluded that both (0001̅) and {11̅01̅} planes are attacked, although possibly at different rates.^10^ However, a more accurate analysis of the etch mechanism reveals that the etching in the direction perpendicular to the {11̅01̅} planes is only apparent.

In reality, we have found that, in a wide range of temperatures and concentrations, the {11̅01̅} planes are very stable in KOH solutions, and the hexagonal pyramids, once formed, can only be attacked from their tops, where a nanoscale -*c* facet is continuously recreated and etched away, as schematically shown in Figure 1. Consequently, by stopping the formation of the (0001̅) top facets from which the etch originates, the {11̅01̅} planes can be fully stabilized.

**Figure 1:**
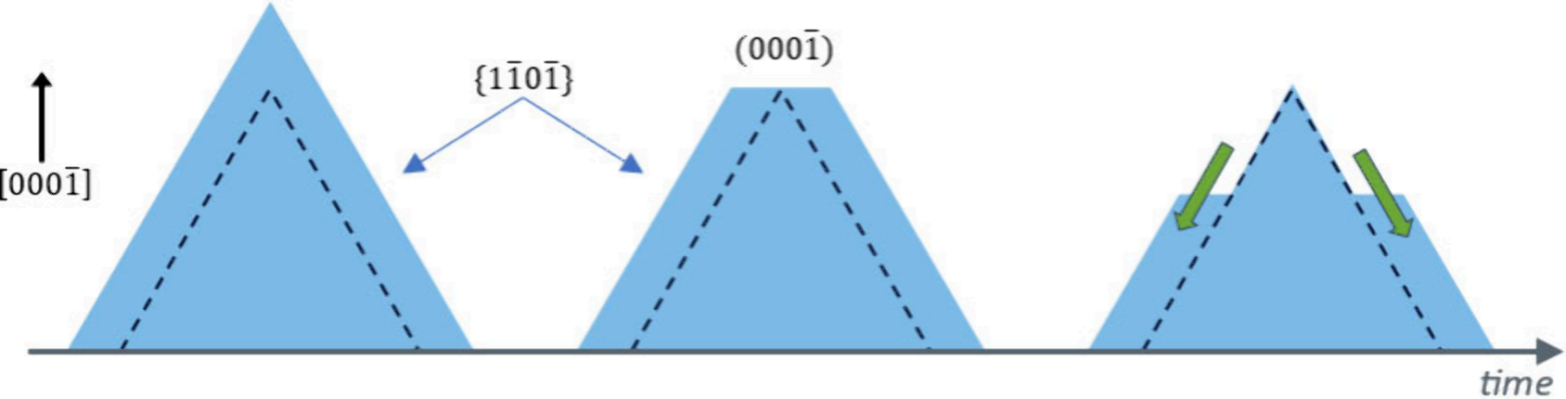
Cross-section time evolution of N-polar GaN pyramids in KOH solutions. The {11̅01̅}-oriented sidewalls are not etched perpendicularly, but attacked from the top, in an atomic layer-by-layer process. As (0001̅)-oriented nanoscale facets of just a few atoms are continuously re-created by the etchant solution, the process continues indefinitely.

This is clearly demonstrated in the scanning electron microscopy (SEM) images shown in Figure 2, where an N-polar GaN sample, first patterned with protective SiN caps and then exposed to a KOH-containing solution, is shown at two different etch times. While the area between the caps did initially convert to a large number of small pyramids of random heights (Figure 2a), the material below the caps was preserved and eventually evolved into a regular array of larger truncated pyramids. Moreover, after meeting with neighboring pyramids, their slanted sidewalls stopped each other’s progression and formed a very stable final configuration (Figure 2b). Hence, this self-limiting behavior also allows for etch-depth control by the choice of appropriate cap diameters and pitch distances. The so-obtained structure is the ideal starting template for a FACELO-like regrowth technique to improve crystal quality of N-polar GaN.

**Figure 2:**
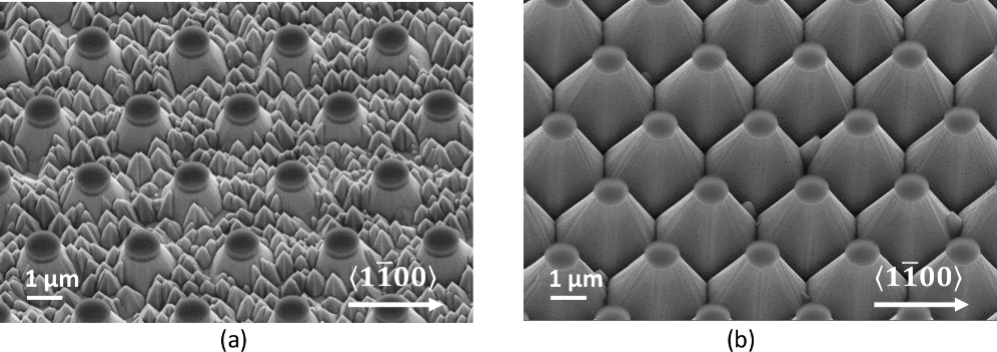
SEM image (45° tilted) of a N-polar GaN sample patterned with SiN caps and etched in AZ 400 K (a) after 15 min at room temperature and (b) after 3 h at 80 °C. While the resulting pyramids are mostly {11̅01̅}-oriented, some deviations from crystallographic planes are induced by the circular caps, particularly at the edges of the sidewalls.

In preparation for the regrowth experiments, we fabricated a set of nominally identical templates similar to the one shown in Figure 2b. The starting planar material was grown in a vertical 3 × 2 in. Close Coupled Showerhead Aixtron MOVPE reactor, using the same approach reported in ref (13), in which a vicinal sapphire substrate, with offcut angle of 4° toward the sapphire *a*-direction, is preconditioned in a high-temperature nitridation step, and the subsequent growth is carried out in a standard two-temperature sequence, although with the nucleation done at a higher temperature, compared to typical Ga-polar growth. In total, ∼2.2 μm of material was grown. Subsequently, ∼100 nm of SiN was blanket-deposited on the wafers using plasma-enhanced chemical vapor deposition (PECVD), and on top of it, a regular array of Pd disks was created by metal evaporation and an optical-photolithography lift-off step. The Pd disks were then used as a hard mask for a dry-etch step to remove all unprotected SiN. Finally, the Pd was dissolved in aqua regia to expose the newly created SiN caps, which had a nominal diameter of 1 μm and were arranged in a hexagonal pattern with a 3-μm pitch distance. Critically, the caps were oriented with respect to the epilayer crystal lattice so that neighboring caps were aligned along the ⟨11̅00⟩ directions. The samples were then wet-etched for 3 h in the developer AZ 400 K, which is a buffered KOH solution, at a temperature of 80 °C to form the patterned templates.

To study how coalescence evolves under different growth conditions, we conducted three series of regrowth experiments. Each series consisted of subsequent runs done under the same nominal conditions but with increasingly longer growth times. In a previous study on Ga-polar GaN nanocolumns,^14^ we found that low-temperature growth in nitrogen favors nanocolumn expansion, while more standard growth in hydrogen and at higher temperatures leads to infilling from the bottom. Based on this observation, we carried out a similar comparison for N-polar growth.

In the first series, samples were grown under nitrogen, at a temperature of 950 °C (determined using a calibrated pyrometer), a chamber pressure of 100 mbar, and a V/III ratio of 1000. As expected, this resulted in a clear expansion of the pyramids and no noticeable infilling of the areas between them. However, as shown in Figure 3, the expansion was not uniform along the height of the pyramids. During the early stages of regrowth, only the tops were significantly affected, while the bottom parts showed minor reshaping. As expansion continued, more and more sections toward the bottom of the pyramids started to follow, which eventually resulted, shortly before full coalescence, in the formation of rather steep sidewalls approaching *m*-plane orientation. An interesting effect of this type of growth evolution is that the pyramid tops, initially fully covered by the SiN caps, expanded outward, forming quite large and crystallographically smooth (0001̅) facets. It is worth highlighting that growth on top of these now perfectly on-axis (0001̅) tops would result in the typical rough morphology observed in non-offcut samples. The fact that they remained smooth for most of the regrowth proves that only lateral expansion was happening in these stages. However, as the growth progressed further, and the lateral expansion became geometrically constrained, the surface supersaturation on the tops started to increase, which initially led to occasional nucleation and formation of sparse hillocks (not shown), and upon full coalescence, to the abrupt onset of the rough surface morphology shown in Figure 3d.

**Figure 3:**
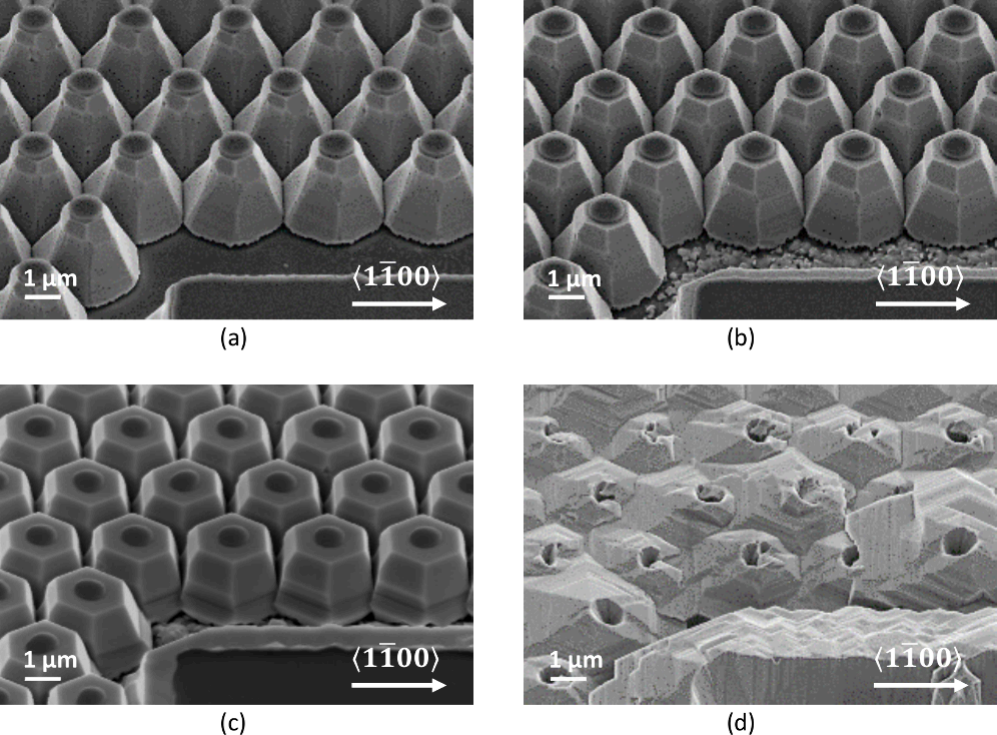
Series of overgrowth experiments conducted in nitrogen at 950 °C. Nominal overgrowth thickness on planar reference: (a) 250 nm, (b) 750 nm, (c) 1750 nm, and (d) 2750 nm.

In the second series of experiments, the samples were grown in a hydrogen ambient atmosphere, at a temperature of 1040 °C, a pressure of 150 mbar, and a V/III ratio of 1000. Compared with growth under nitrogen, the expansion of the (0001̅) tops was much less pronounced, and the areas at the base of the pyramids started infilling from the beginning, as shown in Figure 4. The sidewalls initially expanded maintaining mostly {11̅01̅} orientation, but some flatter facets (most likely {11̅02̅}) eventually formed around the tops (Figure 4b), and became dominant just before coalescence (Figure 4c). This indicates that {11̅01̅} planes grow faster than the {11̅02̅} planes, and faster than the (0001̅) plane, which is the opposite order of what is observed in metal polar-III nitrides.^15^

**Figure 4:**
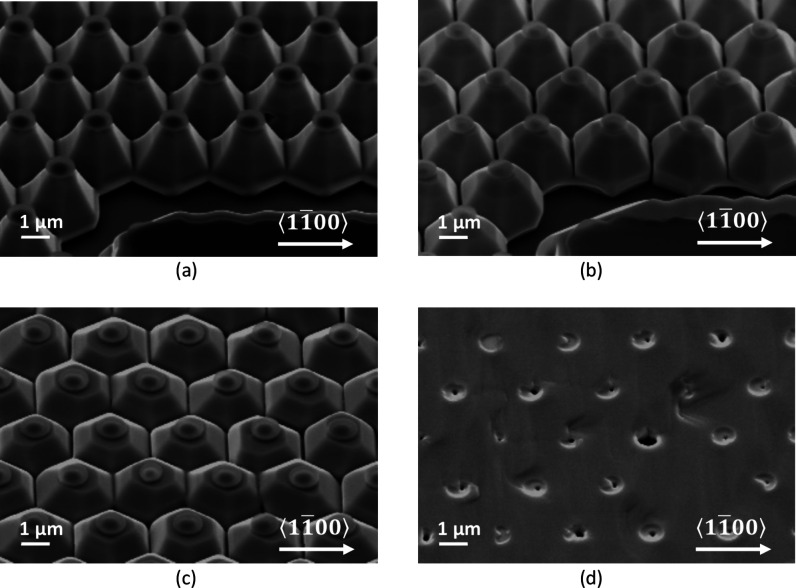
Series of overgrowth experiments conducted in hydrogen, at 1040 °C. Nominal overgrowth thickness on planar reference: (a) 250 nm, (b) 500 nm, (c) 1000 nm, and (d) 2000 nm.

This different type of growth evolution made it possible a gradual coalescence of the pyramids, without the sudden increase of supersaturation on the (0001̅) tops that had led to rough surface morphology on the previous experiment. Most of the areas between the caps became rather smooth, but as the growth progressed even further above the original height of the pyramids, the material that started to grow on top of the caps became rough. In some cases, we even observed Ga-polar nucleation, instead of expansion and overgrowth of N-polar material. This was confirmed by wet-etch in AZ 400 K, which removed all the overgrown N-polar GaN and restored the original geometry of the as-wet-etched templates, but could not dissolve the material grown on top of some of the caps.

However, from the fact that both series of experiments clearly showed no sign of vertical growth on the expanded (0001̅) tops of the pyramids that had formed around the SiN caps, we speculated that the presence of these caps was, in fact, not necessary during overgrowth.

To confirm this hypothesis, a third series of regrowth runs in hydrogen was conducted under the same nominal conditions as before, but on templates whose pyramids had previously been removed of their SiN caps by wet-etching in buffered HF solution. In addition, we increased the number of runs to study the growth evolution in more detail. As can be seen in Figure 5, this time the coalescence phase concluded with the formation of a smooth surface of comparable morphology to that of the original as-grown epilayer. Even at a larger scale, when observed under a Nomarski phase contrast, the surface appeared smooth and free from hexagonal islands or other macroscopic defects, as shown in Figure 5h. Immediately after coalescence, the surface morphology was temporarily dominated by parallel striations oriented perpendicularly to the offcut direction, and whose average distance was exactly half of the pyramid pitch-distance. From this, we can conclude that these striations are formed when the slowly expanding pyramid’s tops finally meet. Being crystallographically (0001̅)-oriented, the tops are necessarily tilted by 4°, with respect to the sample face, which was grown on a vicinal substrate off-axis by the same angle. When neighboring tops meet, they, hence, have slightly different heights, which creates a sort of macroscopic step bunching. However, without any disturbance from the SiN caps, the step edges were free to move as step-flow growth was re-established and eventually redistributed uniformly, restoring a 4° off-axis surface, smooth and free from any major striations.

**Figure 5:**
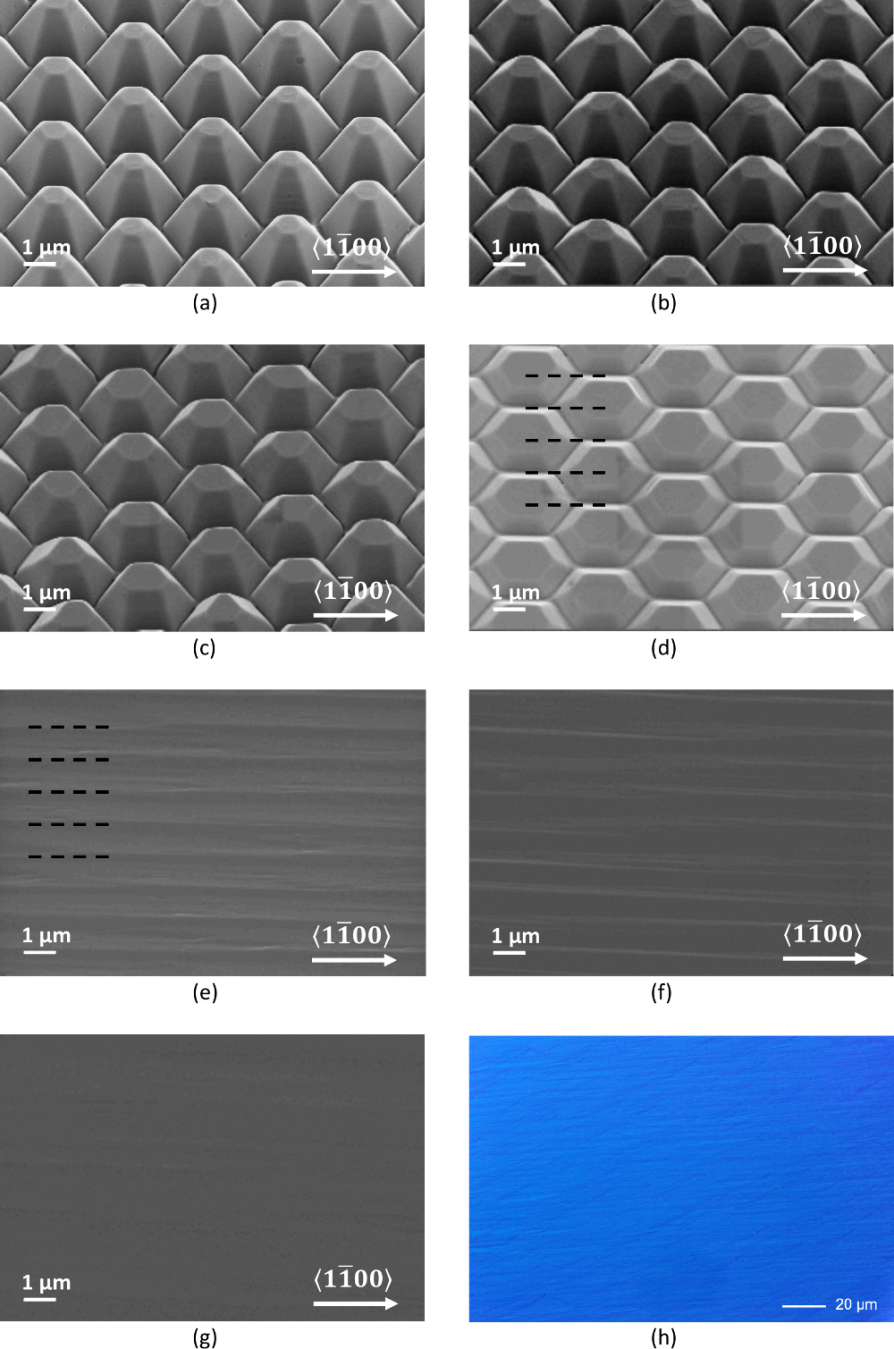
Series of overgrowth experiments conducted in nitrogen, at 1040 °C, on wet-etched templated with removed SiN caps. Nominal overgrowth thickness on planar reference: (a) 250 nm, (b) 500 nm, (c) 750 nm, (d) 1000 nm, (e) 1500 nm, (f) 2000 nm, and (g) 3000 nm. (h) Nomarski microscopy image taken after overgrowth of 3000 nm of material. The formation of the striations and their mutual distance is highlighted by the dashed lines in panels (d) and (e).

In order to gain insight into the evolution of the threading dislocation density during the coalescence phase, the samples of the third series were also analyzed with X-ray diffraction (XRD) using a Malvern Panalytical X’Pert diffractometer. Peak broadening in symmetric (skew-symmetric) reflections is commonly used as a proxy for the density of threading dislocations with a screw (edge) component, respectively.^16^ For this reason, fwhm values of 0002̅ and 101̅1̅ reflections were measured using a PIXcel detector in open-detector mode. At an initial analysis, some fwhm variations were observed at different but nominally equivalent azimuthal angles φ, probably induced by the presence of the large offcut angle at a particular φ. For this reason, the offcut direction, which for all the samples here studied corresponded to a sapphire *a*-direction—and, hence, to a GaN *m*-direction—was defined as φ = 0°. Then, for consistency, all 0002̅ reflections were measured at φ = 90°, and all 101̅1̅ skew-symmetric reflections at φ = 30°.

As shown in Figure 6a, the as-wet-etched starting template had fwhm values of 529 and 552 arcsec, for 0002̅ and 101̅1̅, respectively, which are the same values measured before etching, and similar to those commonly reported in the literature for N-polar GaN films.^1,4,5^ Hence, this sample can be used as a reference to assess the crystal quality improvement due to our technique. As the coalescence phase progressed, the 101̅1̅ fwhm values decreased monotonically, demonstrating a clear edge-dislocation reduction. After regrowth of nominally 3 μm of material, the fwhm decreased to 329 arcsec. For the 0002̅ reflections, a different type of evolution was observed, whereby the fwhm’s initially increased over 650 arcsec, and only after 1 μm of nominal growth, started to decrease and to follow a trend similar to the one observed for 101̅1̅, which demonstrate a comparable reduction also for screw dislocations. At the end of the experiment, the measured 0002̅ fwhm was 321 arcsec. It is worth noting that the final fwhm values here reported represent a rather large underestimation of the crystal quality of the coalesced epilayer as both the newly grown and the underlying original material are probed during these XRD measurements. Nevertheless, the steady fwhm reduction after 1 μm of nominal growth is a clear signature of threading dislocation density reduction in N-polar epitaxial lateral overgrowth. Even though the geometry of the starting patterned template was not optimized, the resulting fwhm values are already significantly smaller than those typically reported in the literature for N-polar GaN on foreign substrates.

**Figure 6:**
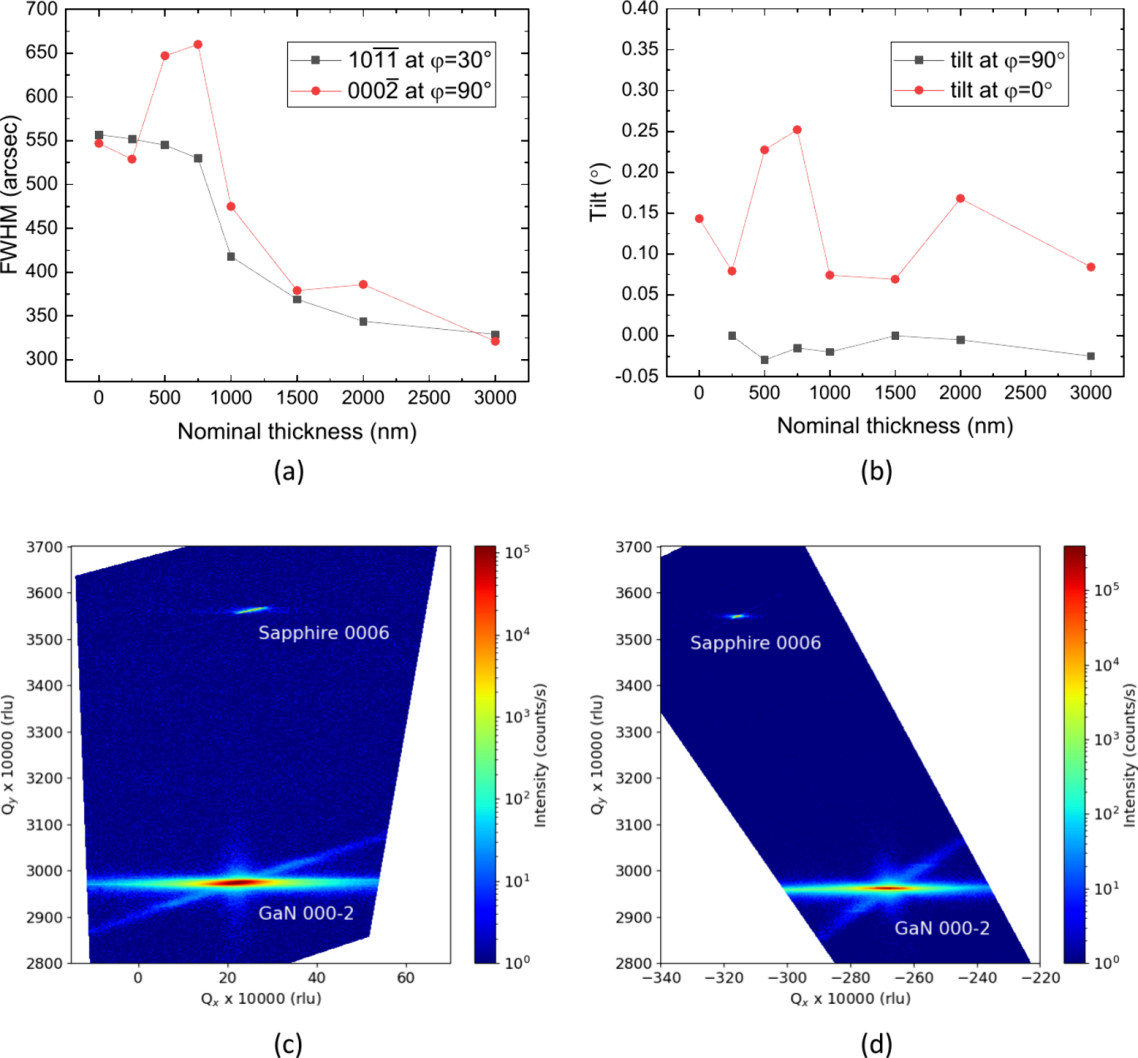
Evolution of the coalescence phase as a function of nominal overgrowth thickness for (a) fwhm values of 0002̅ and 101̅1̅ reflections; and (b) epilayer tilt in the directions corresponding to φ = 90° and φ = 0°. Example of two reciprocal space maps collected, for the same sample, around GaN-0002̅ and sapphire-0006 reflections and (c) φ = 90°, with offcut accommodated by χ, and (d) φ = 0°, with offcut accommodated by ω offset.

In heteroepitaxial growth, lattice mismatch between substrate and epilayer induces strain; however, while in on-axis epitaxy, only *a*-lattice parameter differences matter, for growth on vicinal substrates also *c*-lattice parameter mismatch plays a role. In fully strained epilayers, this is known to cause the so-called “Nagai tilt”.^17,18^ To investigate the presence of any tilt in our samples, we collected a series of reciprocal space maps of the GaN-0002̅ and sapphire-0006 nearby reflections, both at φ = 0° and φ = 90°, as shown for example in Figures 6c and 6d for one of the samples. The epilayer relative tilt α_φ_ at the azimuthal angle φ, was calculated as the offset difference between the two peaks:
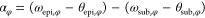
where the subscripts “epi” and “sub” refer to the epilayer and the substrate, respectively. The evolutions of both α_90°_ and α_0°_ tilts are shown in Figure 6b. As expected, there is almost no tilt along the direction φ = 90° perpendicular to the offcut, but a tilt along φ = 0° is clearly noticeable, although of much smaller intensity than Nagai theory would predict, which indicates almost complete epilayer relaxation. Interestingly, the evolution of the tilt in the offcut direction correlates very strongly with the anomalous increase of the 0002̅ fwhm values at the initial stage of the coalescence phase, shown in Figure 6a. This seems to suggest that nucleation of new screw dislocations might indeed be happening at the very early stages of the coalescence, caused either by the epilayer tilt, or, possibly, by its pyramid-to-pyramid variations. However, further work is still necessary to better understand the interplay between macroscopic tilt induced by the growth on vicinal substrates, and microscopic tilt due to screw dislocations.

In conclusion, we have demonstrated—for the first time in N-polar materials—a significant reduction of threading dislocations with FACELO-like overgrowth of a patterned (0001̅) GaN layer. This method represents a promising alternative approach to N-polar epitaxial lateral overgrowth on masked stripes, for which inversion domains have been reported to easily occur,^19^ and that produces films with large differences in crystal quality between masked and unmasked regions.^19,20^ The starting templates were prepared by wet-etching in KOH-containing solution and consisted of a regular array of microscale hexagonal pyramids with sidewalls approximately oriented along the {11̅01̅} planes, and whose positions are controlled by SiN caps previously deposited by photolithography. In contrast to the large, masked stripes mentioned above, our SiN caps can be made as small as desired, which makes the final epilayer highly uniform. The coalescence of so-created pyramids into a final layer of adequately smooth surface morphology required the use of growth conditions that preserved the sidewalls’ orientation over expansion of the (0001̅)-oriented pyramid tops. Although further optimization of the process is still needed, this proof-of-concept demonstration shows that there is room for significant improvement of the current N-polar GaN crystal quality, which would be of great benefit for the development of new and improved N-polar devices.
